# Factors constraining patient engagement in implantable medical device discussions and decisions: interviews with physicians

**DOI:** 10.1093/intqhc/mzx013

**Published:** 2017-02-13

**Authors:** Anna R. Gagliardi, Pascale Lehoux, Ariel Ducey, Anthony Easty, Sue Ross, Chaim M. Bell, Patricia Trbovich, Julie Takata, David R. Urbach

**Affiliations:** 1 Toronto General Hospital Research Institute, University Health Network, 200 Elizabeth Street, Toronto, M5G2C4, Canada; 2 Department of Public Health Administration, University of Montreal, C.P. 6128, succ. Centre-ville Montréal, H3C 3J7, Canada; 3 Department of Sociology, University of Calgary, Social Sciences Building, Room 956618 Campus Place N.W., 2500 University Drive NW, Calgary, T2N2N4, Canada; 4 Institute of Biomaterial & Biomedical Engineering, University of Toronto; 5 Women & Children's Health Research Institute, University of Alberta, 11405 87 Avenue NW, Edmonton, T6G1C9, Canada; 6 Department of General Internal Medicine, Mount Sinai Hospital, 600 University Avenue, Toronto, M5G1X5, Canada

**Keywords:** equipment and supplies, patient participation, health communication, patient rights, qualitative research

## Abstract

**Objective:**

Patient engagement (PE) is warranted when treatment risks and outcomes are uncertain, as is the case for higher risk medical devices. Previous research found that patients were not engaged in discussions or decisions about implantable medical devices. This study explored physician views about engaging patients in such discussions.

**Design:**

Qualitative interviews using a basic descriptive approach.

**Setting:**

Canada.

**Participants:**

Practicing cardiovascular and orthopaedic physicians.

**Main outcome measures:**

Level, processes and determinants of PE in medical device discussions and decisions.

**Results:**

Views were largely similar among 10 cardiovascular and 12 orthopaedic physicians interviewed. Most said that it was feasible to inform and sometimes involve patients in discussions, but not to partner with them in medical device decision-making. PE was constrained by patient (comfort with PE, technical understanding, physiologic/demographic characteristics, prognosis), physician (device preferences, time), health system (purchasing contracts) and device factors (number of devices on market, comparative advantage). A framework was generated to help physicians engage patients in discussions about medical devices, even when decisions may not be preference sensitive due to multiple constraints on choice.

**Conclusions:**

This study identified that patients are not engaged in discussions or decisions about implantable medical devices. This may be due to multiple constraints. Further research should establish the legitimacy, prevalence and impact of constraining factors, and examine whether and how different levels and forms of PE are needed and feasible.

## Introduction

Patient engagement (PE) has been defined as patients (and their families or representatives) and health professionals working in active partnership at various levels across the health care system to improve health and health care [1–3]. The definition recognizes that patients can be engaged at the organizational or system level to improve the design of health services. Examples include, but are not limited to, serving on hospital or government committees ‘to help with everything, from paint chips to policy’ [4] or on panels that develop clinical practice guidelines [5]. The definition also recognizes that patients can be engaged at the level of their own care, sometimes referred to as patient centred care, which is the focus of our study [1–3]. PE in their own care can vary according to patient circumstances and preferences from receiving information or education to being an active partner in the care team by setting goals and taking part in decisions [1]. Methods or tools to implement or achieve PE in their own care include, but are not limited to lay summaries, pre-consultation question prompts, decision aids, shared decision-making and self-management programmes [1–3].

PE in their own health care is desirable because it can improve patient knowledge, relationship with providers, health service use, satisfaction with health care, adherence to recommended treatment and other desirable lifestyle behaviours, and clinical outcomes [6]. However, numerous factors challenge PE. For example, patients may not be familiar or comfortable with PE [7], and physicians may experience role tension between PE and delivering clinical care [8]. Consequently, many patients do not experience desired levels of PE [9], and experts have advocated for improved PE implementation [10, 11].

PE is particularly relevant when treatment-associated risks and outcomes are difficult to predict [1–3]. In such cases, decisions are ‘preference sensitive’ because patients informed of risks and benefits might change their treatment preference [12]. The preference-sensitive concept is germane to the context of non-drug technologies where evidence of the safety and effectiveness of some implantable cardiovascular or orthopaedic medical devices is limited [13, 14], and they have been associated with morbidity and mortality [15, 16]. Medical devices have been defined as non-drug technologies or instruments vital to the prevention, diagnosis, cure or treatment of a disease or abnormal physical condition [17]. Examples of lower risk devices are wheel chairs and endoscopes, and examples of higher risk devices are joint or cardiovascular implants that require invasive procedures.

Few patients appear to have been engaged in medical device decision-making. For example, among Medicare beneficiaries who underwent elective coronary artery stenting in 2008, few reported having discussed treatment options (10%), associated risks (19%) or their treatment preferences (16%) [18]. Among 17 of 22 standardized patients, visits with American cardiologists did not address, or minimized quality-of-life issues and the risks of implantable cardioverter-defibrillator (ICD) placement [19]. All respondents to a survey of American patients who had joint replacement said that benefits were discussed more than risks [20]. In another study of patients undergoing knee arthroplasty in one Canadian centre, most had little knowledge of the risks and benefits of options for devices and surgical approaches, and desired more information [21].

Given the overall benefits of PE, and patient reports of little or no PE for decision-making about higher risk medical devices, further research is needed to explore PE for medical device decision-making. The feasibility of PE is thought to hinge on the effort or resources required from patients, physicians or the health care system [2]. There is a paucity of research from the perspective of physicians on the effort or resources required to engage patients in decisions about medical devices. Such knowledge would provide useful insight on how to better implement PE in this unique context. The purpose of this study was to explore physicians’ views about the feasibility of PE in decisions about higher risk implantable medical devices.

## Methods

### Approach

A qualitative approach was chosen to explore physician views about PE in medical device decision-making [22]. More specifically, the research design employed was based on what is referred to as basic descriptive qualitative research [23]. This method does not generate theory as would other qualitative approaches such as grounded theory technique. Instead, the purpose of basic descriptive qualitative research is to describe factual information about phenomena directly conveyed by participants through semi-structured interviews. It is interpretive and, as we did, often employs a theoretical framework to analyse the findings once generated using methods of qualitative analysis. Rigour and transferability were optimized using standard strategies and reporting criteria [24, 25]. These included exploring responses inductively for emerging ideas, identifying deviant cases, comparison of independent thematic coding across two individuals and demonstrating responses from an array of respondents by including an anonymous identification code with exemplary quotes. Ethics approval for this study was granted by the University Health Network. Participants provided written informed consent prior to being interviewed, in which they were apprised of the research rationale and goals. There was no relationship among members of the research team and participants.

### Sampling and recruitment

Physicians were identified in publicly available certification agency directories, and hospital or university websites, and invited to participate by regular or electronic mail. They were purposively recruited by specialty (cardiac or vascular surgery, interventional cardiology and orthopaedic surgery), region (Canadian provinces), setting (academic, community) and years in practice (self-reported early, mid or late career). A reminder was sent to non-respondents at 2 and 4 weeks from initial contact. Sampling aimed to interview 10 of each specialty who varied by other sampling characteristics. Of 561 physicians invited to participate, 534 declined or did not respond and 27 consented; we were able to schedule interviews with 22 (Table 1).

**Table 1:** Demographic characteristics of interview participants

Physician specialty	Self-reported career stage			Subtotal
	Early	Mid	Late	
Orthopaedic surgeons	10OCE-MB 11OTE-MB 14OTE-AB 15OTE-NS 16OTE-NS 17OTE-NS	06OTM-ON 08OTM-MB 12OCM-BC	03OTL-ON 07OTL-ON 09OCL-MB	12
Cardiac or vascular surgeon, or interventional cardiologists	02CTE-ON 04CTE-ON	01CTM-ON 05CTM-ON 13CTM-MB 19CTM-ON 20CTM-AB 21CTM-ON 22CTM-MB	18CTL-ON	10
Subtotal	8	10	4	22

C, cardiac; O, orthopaedic; T, teaching; C, community; E, early career; M, mid-career; L, late career; two letter code for province.

### Data collection

Telephone interviews were conducted by the principal investigator, a PhD trained Scientist with many years of experience in qualitative research, between 8 April and 28 September 2015. During the interview, participants were asked about factors that influenced their choice of medical devices and their response to adverse medical device events (findings are reported elsewhere). To address the main purpose of this study and elicit views about PE, participants were asked: ‘What is the role of patients in deciding which type of device to use?’ This was distinguished from telling patients about device risks and benefits during the required informed consent process, and described as having a discussion with patients about device options, characteristics and performance. Interviews were audio-recorded and professionally transcribed. Interview length was an average of 30.6 min (median 30.5, range 22.0–45.0 min).

### Data analysis

Analysis was concurrent with data collection, and proceeded until no further unique themes emerged from successive interviews (thematic saturation). Data were organized using Microsoft Office software. The principal investigator identified unique themes using constant comparative technique [22]. First, interview transcripts were read to identify, define and organize themes in participant responses relevant to the main interview questions (first-level coding). Second, a codebook was developed to organize codes reflecting emerging themes, their definition and sample quotes illustrating application of that code. Third, transcripts were reviewed to assess whether and how to expand or merge themes (second-level coding). Saturation was determined through discussion of emerging themes among all members of the research team on three occasions during the iterative data analysis process until it was deemed by consensus that the most recent interviews produced consistent information.

Following qualitative analysis, themes were interpreted in several ways. Level of PE articulated by participants was described according to the Carman *et al*. Multidimensional Framework for Patient and Family Engagement in Health and Health Care [1] as either consultation (patients receive information about their diagnosis and/or treatment), involvement (patients are asked about their treatment preferences) or partnership (treatment decisions are based on patient preferences, evidence and clinical judgement). For example, if participants said that patients were not involved in decisions because it was the physician responsibility to do so, that was categorized as ‘consulting’ with patients. PE processes were described based on the Prokopetz *et al*. commentary about medical devices and shared decision-making. The commentary proposed that it was reasonable for physicians to choose the device best suited to patients, but recommended that they engage patients by providing a rationale for the implant chosen, discussing available evidence in support of the device, disclosing relevant financial relationships, eliciting patient concerns and expectations, and confirming patient understanding [26]. For example, if participants said that a barrier of involvement in decision-making was that patients did not have the capacity to understand technical information, this idea was mapped to the Prokopetz *et al*. recommendation to confirm patient understanding [26]. Feasibility of PE, defined by Grande *et al*. as multi-level factors influencing PE [2], were described based on determinants that emerged from the data. For example, if physicians said that they preferred to use familiar devices on which they were trained, this was mapped to physician factors that constrained PE; and if they said that they used specific devices to fulfil purchasing group contracts, this was mapped to health system factors that constrained PE. Then, multi-level factors influencing PE which emerged from qualitative interviews were tabulated with the corresponding mapped Prokopetz *et al*. recommendations to generate a framework by which physicians can engage patients in decisions about medical devices. All members of the research team met again to review and finalize the interpretation of data.

## Results

### Participants

Twenty-two physicians who implanted cardiovascular (pacemakers, ICDs and stents) and orthopaedic devices (largely hip/knee implants) were interviewed (Table 1). These included 8, 10 and 4 early, mid and late career physicians, respectively, from 5 provinces, and each was from a different hospital. Supplementary Table 1 presents all themes and exemplar quotes. Select quotes are discussed here to illustrate themes, discrepant views and participant characteristics associated with discrepant views.

### Level of PE

Most participants favoured ‘consulting’ patients, described as informing patients about the device that they had already decided to use. One participant said: ‘There is a lot of variability in what different physicians would deem as acceptable consent’ (02CTE), suggesting that patients may receive inconsistent information about medical devices.

Some participants said that patients could be ‘involved’ in particular decisions, for example, choosing a category of device (e.g. tissue or mechanical for cardiovascular, metal or plastic bearing surface for orthopaedic) but not a specific device from within that category.

Few participants, mostly early career physicians, said that patients should be ‘partners’ in medical device decision-making.

It's an important thing in our day and age. This is what's going in them. They have the right to participate. (10OCE)

### Processes of PE

No physicians explicitly stated that they discussed conflicts of interest, evidence for the chosen device, queried patients about concerns or expectations, or confirmed patient understanding. Several participants said that they provided patients with a rationale for choosing a particular device, and discussed the risks and benefits associated with that choice as part of the informed consent process.

I give them a three-page handout that talks in generic terms about the indications, the complications, the success rate, the failure rate, the recovery period. I also say it to them verbally. I tell them that if they have questions let me know. (03OTL)

You need to talk to the patient about what the device is supposed to do, how it is going to be implanted, what risks are there, what potential benefits are there, and all these are outlined in the informed consent as well as verbal discussion. (18CTL)

### Feasibility of PE

Many interacting patient, physician, health system and device/device market factors were said to influence PE. Factors were largely common for disparate types of devices and among physicians with different characteristics. These are summarized in Table 2 along with corresponding recommendations derived from Prokopetz *et al*. for engaging patients in PE [26]. The summary presented in Table 2 provides physicians with a framework of topics by which to engage patients in discussions related to implantable medical devices.

**Table 2:** Factors constraining and enabling PE in decision-making about medical devices

Category	Constraining factors	Enabling factors^a^
Patient	Best fit for physical and demographic characteristics Prognosis (life or death scenario) Age (device longevity greater than expected patient lifespan) Individual desire for PE (most prefer to let physicians decide) Capacity to understand complex, technical information Well informed about manufacturers/devices	Explain why a particular implant is recommended Solicit patient values and preferences regarding unknown risks Probe for other patient concerns and expectations Confirm patient understanding
Physician	Familiarity/comfort with specific device due to training/experience Time required to educate patients	Disclose relevant financial relationships
Health system	Fulfilment of purchasing group contracts Use of least costly device for same indication	Refer patient to another physician who uses a device preferred by the patient
Device or device market	Comparative advantage of different devices for same indication Number of different devices available for the same indication	Discuss available evidence in support of the device Use lay language to distinguish design features and trade-offs between different devices

^a^Constraining factors were mapped to relevant strategies (enabling factors) for shared decision-making in relation to medical devices that were recommended by Prokopetz *et al*. [26].

#### Patient

Some participants said that PE was not pertinent among patients facing death who had no option other than an implantable cardiovascular device, or among older patients for whom orthopaedic devices were likely to last through their lifespan. Several participants said that device selection was largely based on patient physiology and demographic characteristics, and that only the physician could assess these clinical factors to choose the device best suited to each patient.

Many participants questioned patient ability to understand technical information about devices, and said that patients generally wanted physicians to make such decisions. A few acknowledged that this view may be regarded as paternalistic, but emphasized that physicians must be trusted by patients to make decisions in their behalf.

At a certain point it becomes absurd. Are we gonna have to discuss what suture material we use? And why we're using that vendor? The average person is not interested. It's just too heavy for them to grasp. Maybe I'm very paternalistic. I don't think I am. An overarching policy of detailed descriptions of different technologies and why we might use one over the other—I'm really not sure that it's relevant. (21CTM)

Newspapers assume that people can read a grade 5 or 6 level. I think that the general population, to be quite frank, is not smart enough to engage in that discussion. Physicians quite frankly don't have the time to educate people, even in the basics that they would have to know. That sounds really paternalistic, and I should probably apologize for that, but I just can't see that as being workable. That is one of those areas where providers have to make decisions on behalf of their patients, and the patients have to trust their providers to make those decisions in good faith. (06OTM)

These views contrasted with others who said that patients are increasingly well informed, sometimes even more so than physicians.

Patients have researched it a lot on the internet and they know what companies have had issues. I think it's a problem now that there's so much information about these implants out there that the patients can tell me more than I know what instrument companies are good and which are not. (07OTL)

#### Physician

Several participants said that they primarily use devices familiar to them, largely based on their training, to achieve optimal outcomes. Some differing views were expressed. Some participants who use orthopaedic implants said that proficiency with many devices was needed to best meet patients’ clinical needs.

Some people treat everything with one system and some other people treat tailored to the patient. It depends on your philosophy and your training. I'm one of those people that tailor it so I try to look at the patient's issues and find the best solution available. (10OCE)

Some people may feel they're only comfortable sticking with one. But being an arthroplastic surgeon is complex. You need a variety, and there are benefits and down sides to every single implant in terms of correcting for deformities, problems, variations on normal anatomy. In order to give the best outcome for patients, the one or two implants that you're comfortable with may not correct those issues and that's why I feel the need to use a wide variety of implants. (14OTE)

#### Health system

Choice of device was often limited to what was approved for use by purchasing groups at the hospital, regional or provincial level.

There are contractual obligations that would make me try one device more than another. In cases where I can use multiple devices then I would try and fulfill my contractual obligations. (13CTM)

Contractual limitations were viewed as a cost-saving measure that was not necessarily in the best interest of patients.

One of the biggest deciding factors will be cost and not necessarily surgeon comfort, patient anatomy and track record of implant. We've had experience that if you force surgeons to change implants based on a contract that your complication rate goes up for a while. That is problematic when it occurs. So it makes good business sense until you actually go and look at your revision costs over the next months to two years and then, all of a sudden, all of your cost-savings went into pain and suffering of patients and their subsequent care. (08OTM)

#### Device/market

Several participants said that devices were largely interchangeable and a less expensive version of the device was often sufficient, obviating the need for PE.

Surgeons and physicians need to be conscientious about the finances in our health care, you can't be implanting the best of the best in every single person. We have to be selective to some degree. (14OTE)

In contrast, others said that some devices were more advantageous or safe than others, which would support the need for PE.

There are some devices where I'm not switching because it's doing everything I need it to do, and other situations where an iteration of a device provides very helpful advantages in terms of ease of implantation or safety. (21CTM)

PE was viewed as less feasible if there were few devices to choose from on the market.

In the world of implantable ventricular assist devices, there are only two available devices on the market now that are being used predominantly around the world. (04CTE)

## Discussion

In previous research, patients did not achieve desired levels of engagement in discussions about implantable medical devices [18–21]. This study assessed whether and how, from a physician's perspective, it was feasible to engage patients in such discussions. Most participants informed patients about the device they chose, the rationale for that choice and associated risks. Few involved patients in decisions by discussing evidence for the device, eliciting concerns and expectations, confirming understanding or revealing conflicts of interest. None partnered with patients to choose particular devices. Participants described multiple interacting patient, physician, health system and device/market factors that constrained PE, which were common for disparate types of devices and among physicians with different characteristics.

While there is little directly comparable research with which to relate these findings, a number of ideas that emerged from this study have also been identified elsewhere including variability among physicians in the informed consent process, patient preference for physicians to choose devices and the use of devices approved in purchasing group contracts. Interviews with 11 American cardiologists revealed substantial variation in the extent to which they discussed ICD risks with patients [27]. In a survey of 364 American orthopaedic surgeons, cost reduction programmes based on volume discounting at their institution was a frequently listed factor that influenced their decision-making [28]. In the same study, among 102 patients undergoing hip or knee arthroplasty, 93.1% said that their orthopaedic surgeon should choose the device; 5.9% said that physicians should consult patients when making the decision. With respect to PE in general, a systematic review found that time constraints, lack of applicability due to patient characteristics and specifics of the clinical situation were the most frequent barriers of shared decision-making [29].

Our study was unique in that it examined determinants of PE for medical device decision-making. Strengths included the use of rigorous qualitative methods, and analysis of the findings using existing frameworks of PE [1, 2] and PE for medical device decision-making [26]. However, several limitations should be mentioned. The number of participants may appear small, but qualitative research is meant to capture detailed information from few, representative participants. Their views may reflect the Canadian health care setting and may not be transferrable to other settings. However, the devices they use are those used worldwide, and several issues that emerged were also revealed in other studies which support the reliability of these findings [27–29]. To mitigate this, sampling was purposive to capture perspectives from individuals with a variety of characteristics, and achieved thematic saturation which signals that recruitment is sufficient to identify themes, though not necessarily sufficient to explore the characteristics and implications of these themes. This would require larger samples from diverse backgrounds, and would be an interesting future study.

At the patient level, some participants questioned patients’ ability and interest to discuss technical information about devices while others said that patients themselves acquired information about device performance. Research is needed to examine patient capacity to engage in discussions and decisions about various types of medical devices, and interventions that can support PE in this context. The PE literature advocates that partnership is appropriate in all circumstances provided it matches patient preferences about the level of engagement [30]. This study did not interview patients, but other research shows that patient preferences for involvement in device decision-making may vary. For example, in one study, 20 patients who were interviewed about involvement in decision-making for knee implants said that they desired active participation, and 17 said they were not given sufficient information or opportunity [27]. In contrast, in another study, among 102 patients undergoing hip or knee arthroplasty, 93.1% said that their orthopaedic surgeon alone should choose the device [28]. A systematic review of 115 studies of patient preference for involvement in shared decision-making found that the majority of patients undergoing invasive procedures (78.5% across 11 studies) preferred to be involved [31]. This rate was similar to patients with cancer in 43 studies, and higher than patients with other chronic conditions (26 studies) or in the general population (36 studies). Hence, it may behoove physicians to assess patient desire for extent of involvement in decision-making about implantable devices.

However, views among participating physicians about the factors constraining PE raise several implications for practice and for ongoing research. At the physician level, views differed on whether proficiency in one or many devices was ideal. If the latter were true, then surgical mentorship may help physicians to expand their competency in a range of devices such that they could engage patients in a discussion of device options [32]. First, research should establish if patient outcomes differ between physicians who use one or many types of devices.

At the level of devices, participant views contrasted on whether devices were interchangeable. PE is, in part, based on a discussion of the evidence for device safety and effectiveness, however, such information is lacking for many types of devices [13, 14]. The IDEAL (Idea, Development, Exploration, Assessment and Long-term follow-up) framework, originally devised to guide the evaluation of novel surgical techniques, was recently modified to accommodate the evaluation of devices [33]. The IDEAL framework included five stages (first in human, prospective development studies, prospective exploration studies, assessment via RCT or alternatives and long-term study). The IDEAL-D framework includes six stages (first in human, which allows confidential reporting to accommodate intellectual property rights; sequential prospective non-comparative cohort studies to generate insight on operator learning curves and iterative changes to implantation procedures; large uncontrolled prospective cohort studies or audits to build consensus on definitions, quality control and outcome expectations for subsequent trials (for first-of-kind devices, could be omitted for successor devices); assessment via RCT or alternatives (for first-of-kind devices) and long-term study or nested RCTs via device registries). If widely adopted, IDEAL-D processes may lead to greater evidence on the safety and effectiveness of medical devices [33].

At the health system level, and potentially overriding physician, patient and device factors, contractual obligations may restrict physicians from using devices with which they are proficient, or considered best suited for patients’ needs, which was thought to increase costs due to complications and revisions. If this phenomenon is widespread, it challenges whether decisions about medical devices can be considered preference sensitive, which hinges on patients having legitimate treatment options [12]. First, research should establish whether device restrictions imposed by purchasing groups are associated with poor outcomes, perhaps by comparing outcomes at hospitals with and without such arrangements.

In summary, this study revealed several factors that, apart from potentially variable patient preference, challenge PE. Further research is needed to identify conditions, for example, type of device and patient characteristics, in which different forms of PE (consult, involve and partner) are relevant and feasible. For now, most participants agreed that informing and involving patients were feasible. Yet some noted that discussions about device risks and benefits varied across physicians, and this was also found to be true elsewhere [18–21]. This study generated a preliminary framework (Table 2) by which physicians can more consistently and thoroughly engage patients in discussions about medical devices, even when decisions may not be preference sensitive due to constraints on choice imposed by patient, physician, device or health system characteristics. Further research is needed to evaluate use and impact of this framework on patient and physician satisfaction with consultations about implantable devices.

## Supplementary Material

Supplementary Data
