# Supplementary material for: Factors constraining patient engagement in implantable medical device discussions and decisions: interviews with physicians
Source: Int J Qual Health Care. 2017 Feb 13;29(2):276–82. doi: 10.1093/intqhc/mzx013 (PMC5412024; doi:10.1093/intqhc/mzx013)
Supplement: Supplementary Data [file Supplementary_Table1.docx]

Supplementary Table 1. Participant views on patient engagement in decision-making about medical device

| Theme | Cardiovascular devices | Orthopedic devices |
| --- | --- | --- |
| ***No role for patient engagement in medical device decision-making*** | | |
| Physician judgment of best device for patient | The overriding factor is what I think will be the best fit for the patient (21CTM) | I try to look at the patient’s issues and find the best solution available (10OCE) |
| Physician preference based on familiarity | A large part of it is based on what I was exposed to as a trainee, because what you implant as a trainee, that’s comfortable and the nuances of the device are known to you (20CTM) | When I offer surgery I already have in mind which implant I’m gonna use and it’s the one I’m comfortable with (11OTE) |
| Devices are similar | They’re all very similar and there’s no real significant advantage between one and another (01CTM) | We don’t really know a whole lot about how much of a difference there is between implant X versus implant Y (17OTE) |
| Choices are limited to devices in stock | The device selection itself, that’s limited by what we have available (22CTM) | Sometimes we just don’t stock things. We might not carry it in our regular inventory. It doesn’t make sense to carry it if you’re only doing one or two a year (06OTM) |
| Choices are limited to those purchased by hospital or region | There are contractual obligations that would make me try one device more than another. In cases where I can use multiple devices then I would try and fulfill my contractual obligations with the manufacturers which is through our buying group (13CTM) | Sometimes the implant you put in is not what you think is the best for the patient because that’s the only thing available through the buying group (07OTL) |
| Multiple factors influence choice | It depends on the patient’s age, their life expectancy, what options we have available at that point in time – we have certain devices that are available in house, what team members we have to actually implant the device, and the referral centre – what capability they have. It would depend on what we think the progression of care will be in terms of do we use a temporary device and which temporary device do we use, or whether they are appropriate for definitive therapy which would be transplantation. All those factors go into the equation (22CTM) | There’s a bunch of factors. First of all, what’s the best device for the patient? If there’s multiple choices of a similar device then it comes down to cost, accessibility, familiarity for the nursing staff and myself. If it comes down to equality then I’ll just use what we have for ease of use (12OCM) |
| Last resort for terminal or older patients | These patients are facing life or death situations and the device is really their only chance at life so it doesn’t really affect our use of the device (05CTM) | If you’re talking about an older patient whichever device you put in will probably last them (09OCL) |
| Patients not capable | That is so far outside of the technical capabilities of the vast majority of patients (19CTM) | I find it difficult to see how a patient would be able to discern between the types of data that would be generated to different one device from another (15OTE) |
| Patients defer to physicians | We still get the type of patient that expects decision to be made on the basis of my expertise alone (02CTE) | Nine times out of ten they would say whatever you think is best (09OCL) |
| Already addressed by informed consent process | Of course you need to talk to the patient about what the device is supposed to do, how it is going to be implanted, what risks are there, what potential benefits there are, and all these are outlined in the informed consent (18CTL) | Patients have to be given an introduction to it. I tell them what I use, how I do it, I even talk about my surgical approach and I tell them very cursorily about the implants that I like to use (10OCE) |
| ***Patients should be engaged in medical device decision-making*** | | |
| Patient’s right to disclosure | I like to engage people in the decisions because there are a lot of decisions, and a lot of what we do is somewhat grey and so there are some lateral options to consider (02CTE) | I think it’s an important thing in our day and age. The joint registry it out there, it’s accessible to the patients. This is what’s going in them. They have the right to participate (10OCE) |
| For certain decisions | The decision whether they want a mechanical value or tissue valve is in their hands. Once they decide on a category, the type of valve that’s implanted I choose because I use primarily one mechanical and two tissue valves. Depending on their age and if they want a tissue value, it’s one or the other. If it’s a mechanical valve it’s only one choice although we have three on the shelf (20CTM) | I don’t give them the choice of which implant I’m placing except for the bearing surfaces. That’s a choice that I make with the patient. The standard is plastic on metal but if I have a young patient I’ll talk to them about the option of putting in a ceramic on plastic or a ceramic on ceramic because of longevity and I show them the data that’s out there on that and have a discussion with them because there are some downsides. I do it more in younger patients than older (14 OTE) |
| In the context of research | We also have an academic interest in introducing new devices. We’ll say you’re the first one in the world or in Canada to use this and these are the expected risks and benefits for you (05CTM) | If they’re gonna be part of a trial with totally new types of implants then, yes, in that specific setting then maybe it’s worth discussing the potential risk and benefit of going with the old standard versus the new thing (11OTE) |

Unique ID: C cardiac, O orthopedic; T teaching, C community; E early career, M mid career, L late career
